# BILIARY COMPLICATIONS AFTER LIVER TRANSPLANTATION

**DOI:** 10.1590/0102-6720201700020011

**Published:** 2017

**Authors:** Júlio Cezar Uili COELHO, Lucas de Oliveira LEITE, Antonio MOLENA, Alexandre Coutinho Teixeira de FREITAS, Jorge Eduardo Fouto MATIAS

**Affiliations:** 1 Department of Surgery, Clinics Hospital, Federal University of Paraná, Curitiba, PR, Brazil

**Keywords:** Hepatic transplantation, Biliary stenosis, Biliary fistula, Biliary complications, Postoperative complications.

## Abstract

**Background:**

Biliary reconstitution has been considered the Achilles’s heel of liver transplantations due to its high rate of postoperative complications.

**Aim:**

To evaluate the risk factors for occurrence of biliary strictures and leakages, and the most efficient methods for their treatment.

**Method:**

Of 310 patients who underwent liver transplantation between 2001 and 2015, 182 medical records were retrospectively analyzed. Evaluated factors included demographic profile, type of transplantation and biliary reconstitution, presence of vascular and biliary complications, their treatment and results.

**Results:**

153 (84.07%) deceased donor and 29 (15.93%) living donor transplantations were performed. Biliary complications occurred in 49 patients (26.92%): 28 strictures (15.38%), 14 leakages (7.7%) and seven leakages followed by strictures (3.85%). Hepatic artery thrombosis was present in 10 patients with biliary complications (20.4%; p=0,003). Percutaneous and endoscopic interventional procedures (including balloon dilation and stent insertion) were the treatment of choice for biliary complications. In case of radiological or endoscopic treatment failure, surgical intervention was performed (biliodigestive derivation or retransplantation (32.65%). Complications occurred in 25% of patients treated with endoscopic or percutaneous procedures and in 42.86% of patients reoperated. Success was achieved in 45% of patients who underwent endoscopic or percutaneous procedures and in 61.9% of those who underwent surgery.

**Conclusion:**

Biliary complications are frequent events after liver transplantation. They often require new interventions: endoscopic and percutaneous procedures at first and surgical treatment when needed. Hepatic artery thrombosis increases the number of biliary complications.

## RESUMO

**Racional:**

A reconstituição biliar é considerada o calcanhar-de-Aquiles do transplante hepático devido à sua elevada taxa de complicações pós-operatórias.

**Objetivo:**

Analisar os fatores de risco para ocorrência de estenoses e fístulas biliares e os métodos terapêuticos mais eficientes para seu tratamento.

**Método:**

De 310 pacientes transplantados entre 2001 e 2015, 182 prontuários foram analisados retrospectivamente. Foram avaliados o perfil demográfico dos pacientes, tipo de transplante e reconstituição biliar, presença de complicações biliares e vasculares, tratamento utilizado e seus resultados.

**Resultados:**

Foram realizados 153 (84,07%) transplantes hepáticos cadavéricos e 29 intervivos (15,93%). Complicações biliares ocorreram em 49 pacientes (26,92%): 28 estenoses (15,38%), 14 fístulas (7,7%) e sete fístulas seguidas de estenose (3,85%). Trombose de artéria hepática esteve presente em 10 pacientes com complicações biliares (20,4%, p=0,003). Os tratamentos de escolha foram os procedimentos endoscópicos ou percutâneos (incluindo dilatação por balão e colocação de próteses (40,82%). No insucesso deles, foi realizado tratamento cirúrgico (derivação biliodigestiva ou retransplante (32,65%). Complicações ocorreram em 25% dos pacientes que fizeram procedimentos endoscópicos ou percutâneos e em 42,86% dos submetidos ao tratamento cirúrgico. A resolução das complicações biliares foi de 45% com os procedimentos endoscópicos ou percutâneos e de 61,9% com o cirúrgico.

**Conclusão:**

Complicações biliares são frequentes após transplante hepático. Comumente requerem novas intervenções. Os procedimentos endoscópicos e percutâneos são a primeira escolha e os cirúrgicos, empregados na falha destes. Trombose de artéria hepática aumenta a incidência de complicações biliares.

## INTRODUCTION

The first successful liver transplantation was performed by Starzl in the USA in 1967^18^. This transplant was considered a non-experimental procedure by the National Institutes of Health of the USA in 1983^18^. The first successful liver transplantation in Brazil occurred in São Paulo in 1985. The Clinical Hospital of the Federal University of Parana was the first institution to perform this transplantation in the State of Parana in Curitiba in September of 1991^11^. Up to present, a total of 534 liver transplantations was performed in this institution. At the moment, Brazil in the third country of the world in number of liver transplantations performed, after the USA and China^5^.

Biliary reconstruction is considered the Achilles’s heel of the liver transplantation. Biliary complications (BC) are frequent, increase hospital stay, cost, and operative mortality^9^. Quality of life is decreased in patients with these complications due to need of percutaneous, endoscopic and surgical procedures^23^. Even after the advances in transplant patient care and in surgical technique in t he last decades, biliary complications remain the most common postoperative technical complication.

Several risk factors correlate with biliary complications. The most important are hepatic artery thrombosis, acute cellular rejection, liver cold ischemia time, and donor and receptor old age^20^. There are only a few publications that correlate these risk factors with the occurrence of biliary complications in Brazil. 

The objective of the present study was to evaluate the incidence, the risk factors and the treatment of biliary complications after liver transplantation.

## METHODS

This study was approved by the Scientific Committee of Ethics of our institution (#CAAE 50988615.0.0000.0096).

Candidates were selected for liver transplantation by a multidisciplinary committee based on clinical and laboratory data. The waiting list order for transplantation was based on the waiting time up to 2006 and afterwards on the MELD score^14^.

The surgical technique employed for cadaveric and living-donor liver transplantation was described previously^6^ ^-^ ^8^. Venovenous bypass was not employed in this series. Biliary reconstruction was performed after vascular anastomosis. Biliary anastomosis was performed with polydioxanone absorbable suture 6-0 or 7-0, either with separated stitches or with two continuous sutures (anterior and posterior). The knots were tied on the outer surface of the anastomosis. 

Biliary reconstruction was preferably performed with termino-terminal anastomosis of donor’s and receptor’s main bile ducts. In the living donor liver transplantation, the right or the left hepatic duct of the donor was anastomosed to the common hepatic duct of the receptor.

In the presence of more than one biliary duct on the donor, ductoplasty was performed in order to create only one anastomosis between these two joined ducts and the common hepatic duct of the receptor. When ductoplasty was not possible, the two or three bile ducts of the donor were anastomosed to a Roux-en-Y jejunum limb (Roux-en-Y hepaticojejunostomy). In patients with primary sclerosing cholangitis, a Roux-en-Y hepaticojejunostomy was employed primarily. 

Bile duct drainage was not employed. A closed suction drain with two tubes was used routinely. One tube was placed in the subphrenic space and the other in the liver hilum area. For living donor liver transplantation, both the donor and receptor were subjected to magnetic resonance cholangiography. Operative cholangiography was not performed routinely.

Medical records of the patients who underwent liver transplantation were reviewed retrospectively. Patients who did not have complete information were excluded from the study. The analyzed data included type of transplantation, biliary tract reconstruction, presence of vascular and biliary complications, presence of other postoperative complications and method and result of the treatment of biliary complications. The diagnosis of biliary complications was based on clinical, laboratory and imaging exams. The diagnosis of hepatic artery thrombosis was established by Doppler ultrasonography and confirmed by arteriography or angiotomography. 

### Statistical analysis

The values were expressed in mean of simple frequency. The data was evaluated by the Mann-Whitney test, Kruskal-Wallis test and Fisher Test. Differences were considered significant if p≤0.05. 

## RESULTS

Of a total of 326 liver transplantations performed in 310 patients in the period from June 1, 2001 to August 4, 2015, 182 medical charts were evaluated. Of this group, 49 patients (26.92%) had biliary complications after transplantation.

The demographic characteristic of the receptors are shown in Table 1. The age of the receptors varied from six months to 70 years, with an average of 47 years. A total of 127 patients (69.78%) were males and 55 females (30.22%). Cadaveric liver transplantation was done in 153 patients (84.07%) and living donor liver transplantation in 29 (15.93%). All receptors had the same ABO blood group of the donors.

**TABLE 1:** Factors analyzed to evaluate the risk of biliary complications

Factors evaluated	Presence of biliary complications (n = 49)	Absence of biliary complications (n= 133)	p	Total
Mean Age	46.67	47.52	0.3366	
Male	36 (73.47%)	91 (68.42%)	0.5872	127
Female	13 (26.53%)	42 (31.58%)		55
Type of transplantation				
Cadaveric	37 (24.18%)	116 (75.82%)	0.0684	0
Living Donor	12 (41.38%)	17 (58.62%)		0
Biliary reconstruction				
Coledocho-coledochostomy	44 (26.99%)	119 (73.01%)	0.7816	163
Hepaticojejunostomy	5 (29.41%)	12 (70.59%)		17
Hepatic artery thrombosis	10 (20.4%)	7 (5.26%)	0.00383	17
Acute cellular rejection	17 (34.7%)	30 (22.5%)	0.127	47
Hepatocellular Carcinoma	14 (28.6%)	31 (23.3%)	0.5619	45
CMV Infection	5 (10.2%)	9 (6.77%)	0.5339	14

**FIGURE 1:**
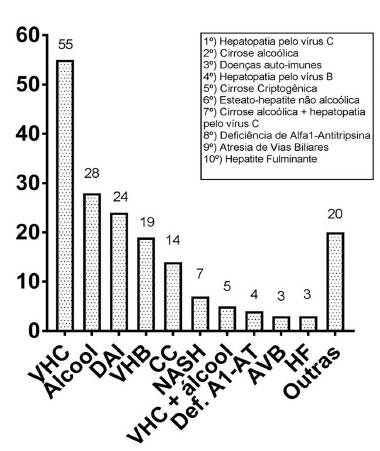
Indications of liver transplantation in absolute numbers

The indications of liver transplantation are shown in Figure 1. The most common indications were advanced chronic liver disease caused by virus C hepatitis and alcoholic cirrhosis, followed by autoimmune disease and advanced chronic liver disease virus B hepatitis. Presence of hepatocellular carcinoma was analyzed separately.

Of 153 cadaveric liver transplantations, 37 (24.1%) had biliary complications: seven fistulas, 26 stenosis, and four stenosis with previous fistula. Of 29 living donor liver transplantations, 12 (41.4%) had biliary complications: seven fistulas, two stenoses, and three stenoses with previous fistula.

The most common biliary complication was stenosis that occurred in 35 patients (19.23%). In seven, the stenosis was preceded by fistula. The diagnosis of biliary fistula was established in the 1^st^ postoperative month all but one patient that was recognized only in the 5^th^ month. Biliary fistula was the most common biliary complication of the living donor liver transplantation. Twenty-five percent of the patients with fistula in this type of transplant had biliary stenosis later. Fistula followed by stenosis was diagnosed in 10.8% of the patients of the group of cadaveric transplantation group.

Four patients with fistula had conservative treatment with good recovery (Table 2). Three were subjected to surgical treatment in the 1^st^ postoperative month: a) two with hepatic artery thrombosis underwent retransplantation and died in the immediate postoperative period of septic shock; b) one had good recovery following Roux-en-Y hepaticojejunostomy.

**TABLE 2:** Methods employed for treatment of biliary complications

Treatment	n	%
Endoscopic treatment	12	24.5
Stenosis	5	10.2
Fistula	5	10.2
Fistula + stenosis	2	4.08
Percutaneous treatment	6	12.24
Stenosis	4	8.16
Fistula	1	2.04
Fistula + stenosis	1	2.04
Endoscopic + percutaneous	5	10.2
Stenosis	5	10.2
Surgical treatment	5	10.2
Stenosis	2	4.08
Fistula	3	6.12
Endoscopic percutaneous + surgical	16	32.65
Stenosis	12 (3 THCP. 2 ERCP. 7 both)	24.5
Stenosis + fistula	4 (1 THCP. 1 ERCP. 2 both)	8.16
Spontaneous resolution	4	8.16
Biliary drainage unknown	1	2.04
Total	49	100

Most patients with fistula were subjected to endoscopic and or percutaneous procedures for diagnosis and treatment (58.8%). These procedures were performed in average in 2.35 times (1 to 5 procedures) in each patient with fistula. The most common procedure used was the endoscopic (in 8 of 10 patients). Fistula resolution occurred in six patients. Complications following endoscopic treatment of the fistula were recorded in three patients (30%): acute pancreatitis, hemorrhage, and acute cholangitis.

Compared to the fistula, biliary stenosis was diagnosed later, in average in the 11^th^ postoperative month. Presence of laboratory exams of cholestasis and magnetic resonance cholangiography findings of dilations and strictures were used to establish the diagnosis of biliary stenosis. Stenosis treatment was performed with endoscopic (n=7) or percutaneous (n=7) biliary dilation. Both endoscopic and percutaneous dilations were used in 12 patients. The average number of dilation was 4.3 (1 to 16 dilations). Resolution of the stenosis was observed in 11 patients: two with endoscopic access, four with percutaneous access, and five with both accesses. A total of 11 patients with no resolution with dilation needed surgical treatment.

Five of 26 (19.23%) patients that needed treatment of the biliary stenosis had complications: infection in two, pancreatitis in two, and migration of the catheter in one. Table 2 shows the methods employed for treatment of biliary complications.

Hepatic artery thrombosis occurred in 17 (9.34%) patients. All them were recognized in the 1^st^ postoperative week. Ten of these patients (58.82%) had biliary complications: six stenosis, three fistulas, and one stenosis preceded by fistula. All biliary stenoses were initially treated with dilation, with an average of 7.8 dilations per patient. Two had endoscopic dilation and five required both percutaneous and endoscopic dilations. The rate of resolution with dilation was 42.86%. Two patients needed surgical treatment: one Roux-en-Y hepaticojejunostomy and one retransplantation. 

Biliary fistula occurred in four patients with hepatic artery thrombosis. One was treated with Roux-en-Y hepaticojejunostomy and two with retransplantation. The presence of hepatic artery thrombosis increased in the incidence of biliary complications in 2.47 times (p=0.0038).

The average MELD score for all patients was 17, similar to the average of patients with biliary complications (17.4). However, the incidence of biliary complications was higher in patients with MELD higher than 25 (2 of 7 patients; 28.5%).

There was no difference of incidence of biliary complications in patients with acute cellular rejection (17 of 47 patients; p=0.127), hepatocellular carcinoma (11 of 45 patients; p=0.5619), hepatitis virus recurrence (6 of 26 patients; p=0.812, Table 1). There was no relationship between the incidence of biliary complications and age above 60 years (p=1.0) or gender (p=0.587, Table 1).

**FIGURE 2:**
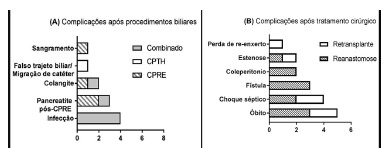
A) Frequency of complications after endoscopic, percutaneous or combinations of these procedures (n=11); B) complications of biliary complications treated surgically with either retransplantation or reanastomosis (n=16)

Note: Patients who presented more than one complication were allocated in more than one category.

Of the 17 patients who underwent Roux-en-Y hepaticojejunostomy, 11 had 

good recovery, three died, two had biliary fistula and one underwent retransplantation (Figure 2). Of the eight patients who were subjected to retransplantation, two had good recovery, one had biliary stenosis e five died.

## DISCUSSION

Biliary complications following liver transplantation are common and increase postoperative morbidity and mortality^1^ ^,^ ^20^. The high incidence is related to the biliary tract vascularization that is exclusively supplied by vessels originated from the hepatic artery^19^. Collateral arterial supply to the biliary tract is suctioned during liver removal for transplantation. Thus, hepatic artery thrombosis causes ischemia and irreversible damage to the intra and extra-hepatic biliary tree^13^.

Other factors associated to biliary complications included acute rejection, iImmunosuppression, ABO incompatibility, cytomegalovirus infection, and technical factors^14^. Acute rejection causes reduction of blood flow and increase of liver volume that predispose to arterial thrombosis. Immunosuppression changes the inflammatory response necessary to healing and formation of normal fibrotic tissue. Cytomegalovirus infection causes vasculitis that reduces hepatic vascularization^8^.

Recently, Nemes et al reported a correlation between biliary complications and elevated MELD score(>25), elevated level of pre-transplant plasmatic sodium, presence of hepatocellular carcinoma, advanced age of the donor (>60 years), prolonged time of anehepatic phase, prolonged time of cold ischemia (>12 h) and duration of stay in the intensive care unit^20^.Some ofthese factors were not evaluated in our study to the lack of complete data in the medical records of several patients.

Of the several potential risk factors evaluated, hepatic artery thrombosis was the only one that was associated with increase in the incidence of biliary complications. The lack of increase of biliary complications in patients with acute rejection, presence of hepatocellular carcinoma, viral hepatitis recurrence, and patient’s age above 60 years observed in this study is possibly due the small number of patients evaluated.

The incidence of hepatic artery thrombosis in this study was 9.3%. Biliary complications occurred in 58.7% of these patients and almost two thirds needed retransplantation. The rate of hepatic artery thrombosis reported in the literature varies from 2.5 to 6.8%^13^.

Our biliary complication rate of 26.9% is similar to other studies^1^ ^,^ ^9^ ^,^ ^13^ ^,^ ^20^. Living donor liver transplantation was almost two times the rate of cadaveric transplantation. In addition to the small size of the biliary duct in the living donor transplantation, some patients have two or more small ducts that need anastomosis.

The explanation for the wide variation of the rate of biliary complications among the transplantation centers was evaluated by some authors. After statistical correction of individual risk factors, Axelrod et al. reported a variation of 75% between the observed and the expected incidence of biliary complications in American hospitals^2^. The possible reasons of the variation include smaller number of transplantations and smaller rate of split liver. It is also important to mention that our public university hospital lacks both specialized personnel and modern equipments.

The most common biliary complication was stenosis, followed by fistula. Healing of fistula may cause biliary stenosis^22^. Biliary fistula occurs earlier than stenosis^15^. In this study, all fistulas were diagnosed in the 1^st^ month post- transplantation, while stenoses were recognized on average on the 11^th^ month.

Biliary stenosis was treated with duct dilation with a balloon, both by endoscopic or percutaneous access. Surgical treatment was indicated in case of endoscopic and or percutaneous treatment failure. Although the primary treatment of fistulas was also endoscopic and or percutaneous interventions, a higher rate of patients with fistula required primary surgical treatment. Retransplantation was needed in patients with hepatic artery thrombosis or after failure of multiple treatments. Our resolution rate of biliary complications by percutaneous and or endoscopic access was similar to other centers^21^.

Our retransplantation rate for treatment of biliary complications was 62.5%, higher than most reports^3^ ^,^ ^4^ ^,^ ^16^ ^,^ ^17^. This is possibly due to limitations of our public hospital, as mentioned earlier. 

## CONCLUSION

Biliary complications are frequent after liver transplantation. These complications require several other endoscopic, percutaneous and surgical procedures that increase the morbidity and mortality and reduce the quality of life of these patients. Hepatic artery thrombosis increases the rate of biliary complications.

## References

[1] Akamatsu N, Sugawara Y, Hashimoto D (2011). Biliary reconstruction, its complications and management of biliary complications after adult liver transplantation A systematic review of the incidence, risk factors and outcome. Transpl Int.

[2] Axelrod DA, Dzebisashvili N, Lentine KL, Xiao H, Schnitzler M, Tuttle-Newhall JE (2015). Variation in biliary complication rates following liver transplantation Implications for cost and outcome. Am J Transplant.

[3] Azoulay D, Linhares MM, Huguet E, Delvart V, Castaing D, Adam R (2002). Decision for retransplantation of the liver an experience- and cost-based analysis. Ann Surg.

[4] Biggins SW (2012). Futility and Rationing in Liver Retransplantation When and How Can We Say No?. J Hepatol.

[5] Bittencourt PL, Farias AQ, Couto CA (2016). Liver Transplantation in Brazil. Liver Transpl Surg.

[6] Carone E, Chapchap P, Porta G, Miura I, Pugliese V, Ayoub A (1998). Transplante hepático com doador vivo familiar. J Pediatr (Rio J).

[7] Carone E, Chapchap P, Pugliese V, Averbach M, Abdalla R, Saad R (1997). Transplante hepático com doador vivo familiar técnica operatória no doador. Rev Col Bras Cir.

[8] Coelho JCU, Matias JEF, Baretta GAP, Celli A, Pisani JC, Yokochi JM (2005). Complicações biliares pós-transplante hepático intervivos. Rev Col Bras Cir.

[9] Coelho JCU, Trubian P, Freitas A, Parolin M, Schulz G, Martins E (2005). Comparação entre o custo do transplante hepático cadavérico e o intervivos. Rev Assoc Med Bras.

[10] Coelho JCU, Wiederkehr JC, Campos ACL (1992). Transplante hepático no Hospital de Clínicas da Universidade Federal do Paraná Descrição dos cinco casos iniciais. Revista Médica do Paraná (Rev Méd Paraná).

[11] Coelho JCU, Habr-Gama A, Gama-Rodrigues J, Machado MCC (2002). Transplante hepático intervivos. Atualização em cirurgia do aparelho digestivo e coloproctologia.

[12] D'Albuquerque LA, de Oliveira e Silva A (1993). Transplante de fígado. Arq Gastroenterol.

[13] Freitas ACT, Coelho JCU, Parolin MB, Matias, Jorge F, Z C (2000). Fatores De Risco E Conduta Nas Complicações Do Trato Biliar No Transplante Hepático. Rev Col Bras Cir.

[14] Freitas ACT, Itikawa W, Kurogi A (2010). The impact of the Model for End-Stage Liver Disease (MELD) on liver transplantation in one center in Brasil. Arq Gastroenterol.

[15] Kadaba R, Bowers K, Khorsandi S, Hutchins R, Abraham A, Sarker S-J (2016). Complications of biliary-enteric anastomoses. Ann R Coll Surg Engl.

[16] Maggi U, Andorno E, Rossi G, de Carlis L, Cillo U, Bresadola F (2012). Liver Retransplantation in Adults The Largest Multicenter Italian Study. PLoS One.

[17] Markmann JF, Markowitz JS, Yersiz H, Morrisey M, Farmer DG, Farmer DA (1997). Long-term survival after retransplantation of the liver. Ann Surg.

[18] Meirelles RF, Salvalaggio P, Rezende De MB, Evangelista AS, Guardia Della B, Matielo CEL (2015). Liver transplantation history, outcomes and perspectives. Einstein (Sao Paulo).

[19] Nacif LS, Ducatti L, Andraus W, Albuquerque LCD (2015). Hepatic Artery Thrombosis after Orthotopic Liver Transplantation. Adv Res Gastroenterol Hepatol.

[20] Nemes B, Gámán G, Doros A (2015). Biliary complications after liver transplantation. Expert Rev Gastroenterol Hepatol.

[21] Park JS, Kim M-H, Lee SK, Seo DW, Lee SS, Han J (2003). Efficacy of endoscopic and percutaneous treatments for biliary complications after cadaveric and living donor liver transplantation. Gastrointest Endosc.

[22] Ribeiro-Jr MA, Medrado MB, Rosa OM, Silva AJ, Fontana MP, Cruvinel-Neto J, Fonseca AZ (2015). Liver transplantation after severe hepatic trauma: current indications and results. Arq Bras Cir Dig.

[23] Testa G, Malagó M, Valentín-Gamazo C, Lindell G, Broelsch CE (2000). Biliary anastomosis in living related liver transplantation using the right liver lobe techniques and complications. Liver Transpl Surg.

[24] Zanchet MV, Silva LL, Matias JE, Coelho JC (2016). Post-reperfusion liver biopsy and its value in predicting mortality and graft dysfunction after liver transplantation. Arq Bras Cir Dig.

[25] Zimmerman MA, Baker T, Goodrich NP, Freise C, Hong JC, Kumer Sean, Abt Peter, Adrian H, Cotterell Benjamin Samstein JEE Development, Management, and Resolution of Biliary Complications After Living and Deceased Donor Liver Transplantation: A Report From the Adult-to-Adult Living Donor Liver Transplantation Cohort Study Consortium.

